# Assessing Fracture Resistance of Restored Premolars with Novel Composite Materials: An In Vitro Study

**DOI:** 10.1155/2021/5512708

**Published:** 2021-08-20

**Authors:** Zahraa Abdulaali Al-Ibraheemi, Huda Abbas Abdullah, Nada Abdlameer Jawad, Julfikar Haider

**Affiliations:** ^1^Department of Conservative Dentistry, Faculty of Dentistry, University of Kufa, Najaf, Iraq; ^2^Department of Conservative Dentistry, College of Dentistry, Tikrit University, Tikrit, Iraq; ^3^Department of Engineering, Manchester Metropolitan University, Manchester, UK

## Abstract

During restorative treatment, premolars restored with resin filling materials using the conventional incremental-fill technique take longer restoration time and undermine the integrity of the tooth. The aim of this study was to assess fracture resistance of premolars restored by various types of novel bulk-fill composite resin materials. Forty-eight (*n* = 48) freshly extracted sound maxillary first premolars were used in this in vitro study. The teeth were divided into six groups, each having 8 specimens. Group A (positive control) was allocated for the intact teeth. For specimens in Groups B to F, a large cavity (Class-II MOD) was prepared with a standardized dimension of cavity (3 mm depth on the pulpal floor, 4 mm at the gingival seat, and 3 mm cavity width). Group B represented prepared teeth without any restoration. Group C, Group D, Group E, and Group F were restored with Tetric EvoCeram^®^ incremental-fill (conventional), Beautifil bulk-fill, Filtek posterior bulk-fill, and SonicFill 2 bulk-fill restorative materials, respectively. All samples were finished and polished with an enhanced finishing kit and stored in distilled water for a month before the fracture resistance testing. All the samples were exposed to the axial loading (the speed of crosshead was 1 mm/min) in a computer-controlled universal testing machine (LARYEE, China) via a steel bar (6 mm in diameter) and the maximum applied force in Newton was recorded as the fracture resistance. One-way analysis of variance (SPSS 21) was used to compare the fracture resistance within the groups, and Tukey's post hoc test was used to determine the difference between the groups. The lowest value of fracture resistance was recorded for Group B, and the highest value was recorded for Group A followed by the values of Group D, Group C, Group F, and Group E. One-way ANOVA revealed a statistically significant difference between the groups (*P* < 0.05). Nonsignificant difference was found between the premolars restored by bulk-fill and conventional composites. Among the bulk-fill restored specimens, Beautifil restorative demonstrated significantly higher fracture resistance in comparison with the other two bulk-fill restored specimen groups (SonicFill 2 and Filtek). Bulk-fill composite such as Beautifil could be an alternative option to conventional incremental-fill composite for premolar restoration.

## 1. Introduction

Modification or damage in structure of the tooth due to trauma, dental caries, and endodontic and restorative procedures has an undesirable effect on its fracture strength and increases the risk of cusp cracks and fracture. Cusp fractures commonly occurring in premolars teeth under occlusal load due to the undesirable ratio of the crown to root, anatomical form, and exposure to compressive and shear forces [1, 2]. Together, the Class-II mesio-occluso-distal (MOD) cavity in maxillary premolar creates a particular challenge for the filling materials in concern of resistance to tooth fracture and longevity. Therefore, the damaged posterior teeth need to be filled with a restoration material that is capable of withstanding fracture when receiving a great amount of occlusal pressure. Composite resin materials are appropriate alternatives to amalgam in the posterior teeth at stress-bearing areas and significantly increase the fracture strength of the dental structure due to its ability to reinforce the tooth by bonding the restoration material to the tooth structure and may act as an internal splint to further stabilize the teeth [3].

One of the intrinsic features of the composite resin materials is the polymerization shrinkage (PS). In general, the incremental placement technique is employed to minimize the PS [4]. However, this placement technique has certain drawbacks associated with the restoration time particularly for wide cavities where voids may be entrapped between layers [5]. New classes of dental restoration products have been developed to overcome these challenges associated with the incremental technique by simplifying the procedures resulting in the reduced number of restoration steps and shorter clinical time.

The novel class of high- and low-viscosity resin nanocomposites has the ability to place in increments of up to 4 or 5 mm in Class-I and Class-II restorations [6, 7]. However, gap formation and shrinkage at the higher depth cavity still remains challenging issue for bulk-fill resin composites [8]. Among them, Filtek bulk-fill Posterior Restorative (3M ESPE, USA) is a notable one having two innovative methacrylate monomers that work in combination with each other in lowering polymerization stress. SonicFill 2 (Kerr Corp., USA), according to the manufacturer, has high filler loading of up to 81.30 wt%. Sonic vibration is applied via a special handpiece to decrease the viscosity and simplify the composite placement procedure [9]. Beautifil bulk-fill restorative (Shofu, Japan) is classified as multifunctional giomer composites and produced with a complex balance by combination of fillers with dissimilar types of monomers to reduce shrinkage and stress associated with the polymerization process. The surface prereacted glass fillers of Beautifil bulk-fill restorative have exceptional surface treatment to enhance the wettability and integration to the matrix.

Although many studies have been conducted on assessing the fracture resistance of different types of bulk-fill composites as premolar restoration materials [10], further understanding is still required on bulk-fill deep and large restorations with new generation nanocomposites. The aim of this study was to test the fracture resistance of maxillary first premolar restored using multiple types of bulk-fill composite resin materials. The null hypotheses of the study are as follows:

1. There would be nonsignificant difference in the resistance to fracture of sound teeth and all restored teeth
2. There would be nonsignificant difference in the resistance to fracture of specimens restored by multiple types of conventional and bulk-fill composite materials
3. There would be nonsignificant difference in the resistance to fracture of specimens restored by different bulk-fill composite materials

## 2. Materials and Experimental Methods

### 2.1. Sound Teeth Extraction and Preparation

Forty-eight (*n* = 48) sound teeth (maxillary first premolars freshly extracted for orthodontic treatment reasons and instantly stored in distilled water) were assembled for this in vitro study. They were thoroughly cleaned with periodontal air scalar (Victor C9000, Taiwan) and polished with pumice paste (Master-Dent, USA) for removing soft tissue remnants, calculus, and residual plaque. The teeth were nearly similar in morphology and size and were free from caries and cracks as observed during visual inspection. Maximum standard deviation was less than 10% from determined mean of bucco-palatal width (BPW) of the selected teeth as measured using a Vernier caliper. Then, roots of each premolar were inserted in acrylic resin (Vertex, the Netherlands) filled premade silicon mold up to 2.0 ± 0.5 mm marked above the cemento-enamel junction. This will create a flat base for cavity preparation, tooth restoration with resin composites and conducting fracture tests.

### 2.2. Specimen Grouping, Cavity Preparation, and Restoration

The prepared tooth specimens were randomly distributed into six groups (*n* = 8) as presented in Table 1. Unprepared sound teeth were used as the positive control and the prepared teeth with Class-II cavity was used as the negative control. Group C was conventionally filled with Tetric EvoCeram and the other groups were filled with three bulk-fill restoratives. The details about the material can be found in the study by [11].

**Figure 1:**
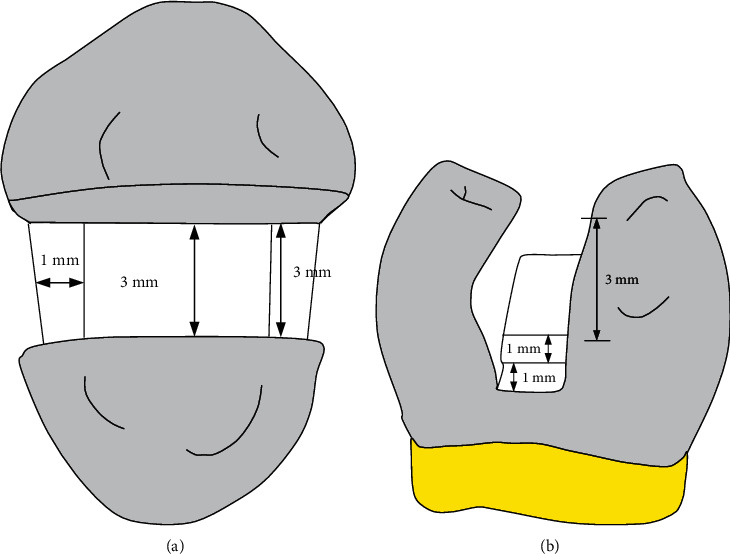
Schematic diagram showing tooth cavity dimensions: (a) occlusal view and (b) proximal view.

For the specimens from Groups B to F, a large cavity (Class-II MOD) were prepared using a hand piece (high-speed) and a flat-ended, parallel-sided diamond fissure bur (Microdent, China) under continuous flood cooling by water. The cavity dimension was standardized with a nominal 3.0 mm depth from the cavo-surface (CS) margin to the pulpal floor, 4.0 mm to the gingival seat, and 3.0 mm width. The internal line angles of the cavity were rounded, and cavity margins (CS margins) were prepared at 90° as shown in Figure 1.

For the specimens from Groups C to F, the transparent plastic SuperMat^®^ band was applied before each restorative procedure and Scotchbond™ Universal Adhesive (3M ESPE, USA) was used as per the instructions provided by the manufacturer in etch and rinse modes. The etching procedure was carried out by treating each cavity with a super etchant (phosphoric acid gel, 37%) for 15 seconds, rinsed thoroughly by water for 30 seconds to ensure removing of the etching agent and dried gently with air stream for 2 seconds to remove excessive water and to avoid dentin dryness. Then, the bonding agent (Single Bond Universal) was applied and cured for 20 seconds with an LED light cure device (Perfection Plus Ltd, UK).

For Group C, Universal Tetric EvoCeram composite (Shade A2) was used to restore using wedge-shaped layering technique. The first layer was applied beside the gingival seat of proximal boxes and palatal wall and cured with another layer applied beside the buccal wall and cured. This procedure was repeated for the occlusal part until the cavity was completely filled and a total number of increments reached eight for every tooth. Each increment was cured for 20 seconds with a Perfection Plus curing device (Power Intensity, PI = 800 mw/cm^2^) according to the manufacturer's instruction.

For Group D, Beautifil bulk-fill composite (Universal Shade) was used for the restoration. In accordance with the manufacturer's directions, it was placed into the preparation as a single layer reaching to approximately 4 mm and cured for 20 seconds with a Perfection Plus curing device (PI = 800 mw/cm^2^). Moreover, it was cured from the buccal and lingual side for an additional 20 seconds.

For Group E, the predosed capsule of Filtek Bulk Posterior Restorative (Shade A2) was loaded into a gun and applied as per the manufacturer's suggestions from the tip of the capsule by injecting the material into the deepest portion of the cavity. The tip was gradually withdrawn until the cavity was filled up as a single step and cured in the same way as in Group D.

Group F was restored by SonicFill 2 composites (Kerr Corp., USA) as per the manufacturer's suggestions. A unidose tip of the SonicFill 2 composite was inserted into the SonicFill™ hand piece by pushing the plunger back into the hand piece and maintaining moderate pressure on the tip, and the hand piece was rotated in a clockwise direction until the tip was screwed into place. As the hand piece was turned on by 3° to restore cavity, the viscosity of the SonicFill 2 was changed by sonic activation to a lower viscosity. The teeth were restored by a steady, continuous stream, keeping the tip below the composite surface, and the tip was withdrawn as the cavity was completely filled and cured in the same way as in Groups D and E.

**Figure 2:**
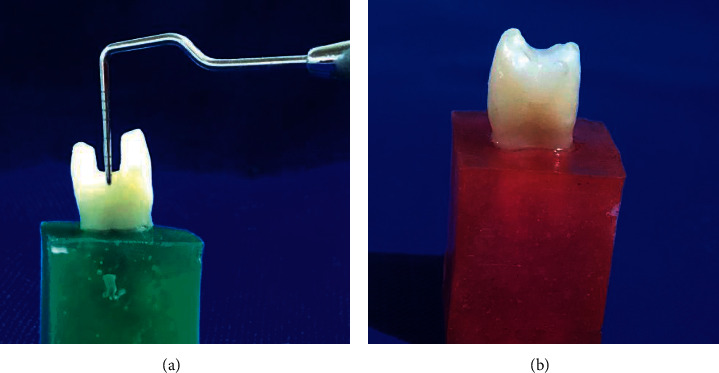
(a) Tooth sample with cavity and (b) restored tooth.

Finishing and polishing of all composite-filled tooth specimens were conducted using an enhanced finishing system as per the manufacturer's suggestions. The finishing was started 10 min after the final curing. These specimens were kept under distilled water (37°C) for 1 month before the mechanical testing.  Figure 2 presents a sample tooth with the cavity and a restored tooth.

### 2.3. Mechanical Testing

**Figure 3:**
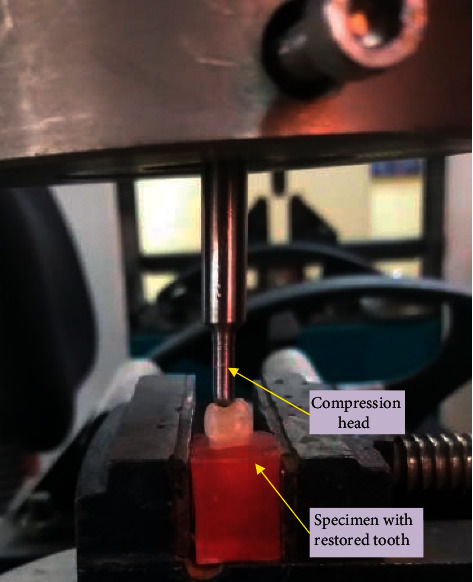
Mechanical testing in a universal testing machine (LARYEE, China).

The specimens were exposed to loading along the axial direction in a computer-controlled universal testing machine (LARYEE, China) via a specially made steel bar (6 mm tip diameter) centered over the occlusal surface with cusp inclination as shown in Figure 3. Special care was given while positioning the compression head on the sample in order to avoid any variations during testing. The speed of crosshead was 1 mm/min and the capacity of the load cell was 50 kN. Load was continually applied until the specimens fractured and the force in Newton was documented. The peak force required for fracturing the specimens was considered as the fracture resistance (FR). The tests were performed by the same operator by following a standardized method in order to maintain consistency during testing.

### 2.4. Failure Analysis

Failure modes of the specimens were assessed and categorized as cohesive, adhesive, and mixed mode of failure. Cohesive type of failure represented the fracture that occurred within the bulk of the tooth structure or restoration (without exposure of any adhesive layer), while adhesive type represented the fracture at the interface between resin and the tooth. The mixed failure included a mixture of both cohesive- and adhesive-type failures [9, 12]. Failure mode was identified by observing the fractured specimens under an optical microscope. The number of different types of specimen failure in each group was recorded, and their percentages were calculated.

### 2.5. Statistical Analysis

The mean fracture resistances with standard deviations (SDs) of the specimens in all groups were calculated. One-way analysis of variance was conducted using IBM SPSS 21 software to compare the fracture resistance of the groups and Tukey's honestly significant difference (HSD) post hoc test at a 95% significance level to determine the difference between groups.

## 3. Results

### 3.1. Fracture Resistance

**Figure 4:**
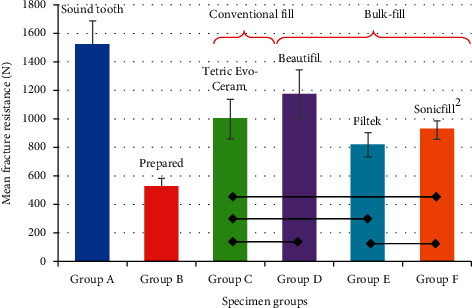
Mean fracture resistance (*N*) different specimen groups. Horizontal bars joining two groups indicate nonsignificant difference (*P* > 0.05).

Fracture resistance (FR) of the premolars in each of the six groups (mean ± SD) is presented in Table 2 and Figure 4. It was clear from the results that the sound teeth always had significantly higher FR than any restored teeth. Conventionally filled group did not show any significant difference compared to the bulk-fill groups. However, among the bulk-fill groups, the Beautifil group showed significant difference in comparison with the other two bulk-fill groups. However, no significant difference was observed between the Filtek and SonicFill 2 groups.

ANOVA of different groups are presented in Table 3. Highest mean FR was observed with Group A sound teeth (1525.38 N) followed by Group D, restored by Beautifil bulk-fill posterior restorative (1172.75 N), Group C restored by universal Tetric EvoCeram (998.75 N), Group F restored by SonicFill 2 bulk-fill composite (921.50 N), and Group E restored by Filtek bulk-fill (817.75 N). However, the lowest mean FR value was observed with Group B specimens prepared without any restoration materials (513.13 N). The One-way ANOVA test reported a highly significant variance among the six groups (*P*=0.000).

On intergroup comparisons, Tukey's post hoc test, Group A had the highest FR (1525.38), which was significantly a higher value than the other five groups (*P* ≤ 0.000) as shown in Table 4. Group B had the lowest FR mean value (513.13 N), which was significantly lower than all the other five groups (*P* ≤ 0.000). Among the restored teeth, there was a statistically significant difference in FR between Group D and Group E (*P*=0.000), as well as between Group D and Group F (*P*=0.003). However, there were no differences between the other restored groups.

### 3.2. Modes of Tooth Failure

**Figure 5:**
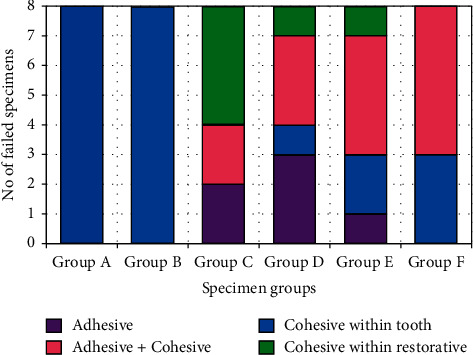
Distribution of tooth failure modes in the specimens from different groups.

**Figure 6:**
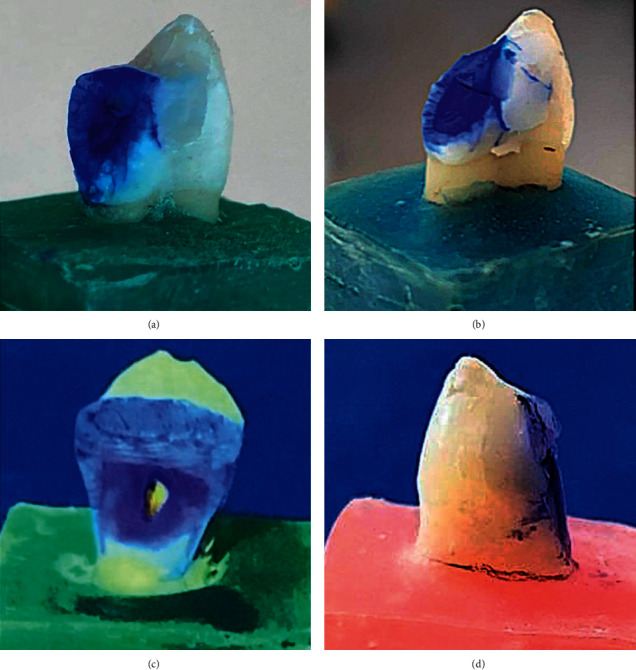
Failed specimens: (a) cohesive within tooth, (b) mix of adhesive and cohesive, (c) adhesive, and (d) cohesive within restoration.

Figure 5 presents the number of specimens failed in each group in four different modes. Only cohesive failure was observed in the sound and unrestored teeth. However, in all the restored teeth, a mixed mode of failure with a combination of purely adhesive, adhesive + cohesive, cohesive within tooth, and cohesive within restorative was appeared. In the conventionally filled group (Group C), the cohesive within restorative mode was dominant, while the adhesive + cohesive mode was dominant in the Filtek and SonicFill 2 bulk-fill groups. However, both adhesive + cohesive and adhesive modes were dominant in the Beautifil restored group. Examples of cohesive within the tooth and a mix of cohesive and adhesive failures are shown in Figure 6.

## 4. Discussion

The polymerization shrinkage and their associated stress is a major factor that governs the success of the restoration using composite resins. Stresses are generated both within the composite resin and the tooth structure due to the contraction during the polymerization process and thus forms microcracks in the restoration, tooth, and at the tooth-composite interface when the occlusal forces are applied. These microcracks can propagate the fracture in tooth or marginal gap formation and consequently leads to restoration failure [13]. The layering technique is considered as the standard technique for dental resin composites placement, which helps in reducing polymerization shrinkage and stress by allowing enough light penetration for adequate polymerization in order to overcome the complication of insufficient curing beyond a certain depth. In spite of these advantages, the layering technique has several disadvantages such as lack of bonding between the layers, the possibility of void incorporation or contamination, and the need for more placement time [14]. Therefore, bulk placement technique was used with the introduction of a novel type of resin called bulk-filling posterior composites. This class of composite allows to place restoration materials in increment up to 4 mm thickness and cured as a one layer. This could be due to the addition of photo-initiators and improvement of filler characteristics of bulk-filling materials [15, 16].

In this study, maxillary first premolar was used because they are more susceptible to cusp fractures due to their unfavorable anatomical shape, crown-root ratio, and crown volume [17]. Furthermore, the premolars are uniform in size, form, shape, and mostly the common extracted sound tooth for the orthodontic treatment [18]. The specimens were kept in distilled water (37°C) for 1 month until testing to give enough time for the composite resin to reach a state of equilibrium of water sorption [19].

In this study, the first null hypothesis, which stated that there would be a nonsignificant difference in FR values of the intact and restored premolars, was rejected as the FR values of the sound teeth were significantly higher than both the prepared teeth and restored teeth. This might be attributed to the presence of a continuous circle of dental structure composed from buccal and lingual cusps and intact marginal ridges. While the teeth with Class-II MOD cavity undergo a greater reduction in fracture resistance as a result of the damage at the marginal ridge, which weakens the residual tooth structure and increases their susceptibility to fracture [20–22]. This came in general agreement with other studies which reported that the intact teeth displayed a greater value of FR when compared to the prepared unrestored teeth [5, 20, 23, 24]. The second null hypothesis, which stated that there would be nonsignificant difference in FR value of premolar restored with bulk-fill restorative materials and that incrementally restored with conventional composite was accepted. This result was also in agreement with other studies, which reported that nonsignificant variances between the novel bulk-fill composite placed as single layer and incrementally placed nanocomposite [6, 25–28]. Recent articles have also drawn the same conclusion after reviewing the relevant literature on different types of restorations, tooth restored, and restoration techniques [29–31].

The third null hypothesis, which stated that there would be nonsignificant difference in FR values of premolar restored with various types of bulk-fill composite materials, was rejected. The mean FR value of the specimens restored by the Beautifil bulk-fill restorative material were significantly different compared to the other two types of bulk-fill composites. This might be attributed to higher filler loading in Beautifil composite (87% wt, 74.5% vol.), which resulted in increased composite stiffness with higher modulus of elasticity and consequently led to a greater fracture resistance [28, 32, 33]. Abdulhameed et al. also found similar results in their study, which reported that a Beautifil bulk-fill restorative had significantly greater value of fracture resistance than nano hybrid (Tertic EvoCeram) and nano-filled (Filtek) [34]. However, other study disagreed and reported that the values of FR of the high-viscosity bulk-fill giomer are statistically lower than both high-viscosity bulk-fill as well as the incrementally placed nanocomposite [15]. The authors argued that high filler content could lead to inadequate light penetration diminished the degree of conversion and consequently resulted an incomplete polymerization process [35]. Shrinkage and voids during restoration in both conventional and bulk-fill composites are the major issues. In a recent study, it was found that by applying a thin or thick layer of flowable liner in an incremental way underneath Tertic EvoCeram bulk-fill restoration could act as a stress reliever [36]. In another study, Micro-CT was employed to evaluate the internal void in different bulk-fill composite restorations with different types of insertion techniques. Preheating insertion technique was found to be effective in significantly reducing the void percentages [37]. Marginal integrity was assessed for tooth restoration with high viscosity and flowable bulk-fill composites and conventional composite by classifying tooth-composite as continuous, noncontinuous, or not judgeable at 20 kV and 200× magnification in a scanning electron microscope both before and after thermo-mechanical loading. The high viscous composite showed similar or better marginal integrity than the conventional resin [38]. Better color stability was also reported in a 6-year clinical trial with a bulk-fill composite compared to an incremental-fill composite [39].

In this study, nonsignificant difference in FR values (*P* > 0.05) was observed between the groups restored with bulk-fill Filtek and SonicFill 2 composites. This observation was aligned with other study reported in the literature [40]. This could be attributed to having nearly the same reduced polymerization shrinkage according to the manufacturer information (Filtek by 1.39%; SonicFill 2 by 1.6%) and also thanks to the specific modulation to reduce the polymerization shrinkage. Filtek bulk-fill posterior restorative is based on a true nano-filler technology and two innovative methacrylates and 1,12-dodecane dimethacrylate (DDMA), which act as a stress reliever and allow the network to re-arrange and get adapted through and/or after the polymerization. This procedure gives relaxation property for the developing network and subsequently relieves the internal stress [5, 40, 41]. In addition, replacement of glass fillers with zirconia/silica fillers improves fracture toughness [5]. While the SonicFill 2 contains rheological modifiers, which permits the particles to move freely resulting in a severe reduction of viscosity up to 84% under sonic activation. This process improves pre-gel stress-relief through internal flow and reduces the polymerization shrinkage stress to 1.6% [42]. Furthermore, SonicFill 2 provides greater adaptation to the cavity walls, and thus reducing the size and incidence of voids located along the line angles and the margin of the cavity and minimizes gap formation. Therefore, the possibility of cracking could be reduced to achieve enhanced fracture resistance [40, 43].

Regarding the failure mode, Group C showed adhesive failure type in 25% of the specimens, cohesive type of failure in restoration in half of the specimens (50%) and the remaining 25% revealed a mixed (cohesive and adhesive) failure type. This result might be attributed to the presence of voids or contamination between composite increments. These voids could be formed due to the porosity of the resin that comprised of oxygen and produced inhibiting zone leading to bond failures between the layers [43]. Group D reported adhesive type of failure in 37.5% specimens, cohesive failure within the tooth structure in 12.5% specimens, and cohesive failure within the restoration in 12.5% cases and mixed type of failure in rest of the 37.5% cases. Again, higher filler loading (87%) leading to high modulus of elasticity in the Beautifil composite could be reasoned for the small percentage of cohesive failure within the restoration. The applied load transmitted to the tooth restoration interface could cause the adhesive and mixed types of failure [26]. Group E showed failures related to both adhesive and cohesive within restoration for 12.5% specimens, cohesive type of failure within tooth structure in 25% and mixed type in 50% specimens.

Group F showed 37.5% cohesive type of failure within the tooth structure and 62.5% mixed type of failure. This could be attributed to adaptability of the composite to the walls of cavity without entrapping void. The fluctuation of viscosity in addition to great fracture toughness and lower polymerization shrinkage enhanced the ability of the filling to absorb the applied load and maintained the interface intact between the tooth and the restoration [43]. The cohesive type failure within the composite resin could indicate the longevity of the restoration. From this point Group F is far better than the other ones as no such failure was observed in this group (Figure 4). The other two bulk-fill composites (Group D and Group E) also showed small number of cohesive failures within the restorative (one specimen each) compared to the conventional fill composite (four specimens in Group C). To simplify the detailed failure classification used in this study, other simple classification (favorable and unfavorable failures) was also used to assess the fracture resistance of the restored teeth [44–46].

Even though one single operator conducted all the preparation and filling, careful attention was given in conducting the compression tests, there still might be some small variations, which could not be avoided, and this might affect the results. Furthermore, the premolars were selected after careful examination and measurement, but still some natural variations in structure, morphology, and strength among the teeth might have some influence on the results. As this study was performed in vitro, the results could vary under natural oral environment condition such as temperature and saliva. The constant forces applied in one direction during the fracture tests also did not fully mimic the loading condition in the oral environment during mastication [45]. This study provides a baseline for future in vivo studies, where clinical restoration can be carried out with the best bulk-fill restorative in order to assess the success of the treatment through patient's feedback during and after treatment.

The new bulk-fill composites obviously have benefits over conventional composites in clinical applications because they tend to reduce the processing time and simplify the restoration process without any significant impact on the fracture resistance of the restored teeth even though FR of sound tooth will always be higher than the restored tooth. In addition, the bulk-fill composites could enhance longevity of the restoration to a certain extent.

## 5. Conclusion

In this study, a comparison was made in the fracture resistance (FR) of premolars restored with different composite resins under conventional incremental-fill and bulk-fill modes. Within the limitations of the study, it can be concluded that no significant difference was found in FR between the conventional and bulk-fill restorations. When only the bulk-fill composites are considered, Beautifil produced the best restoration in terms of both FR and longevity (indicated by lower number cohesive within restorative) in comparison with the other two bulk-fill restored specimen groups (SonicFill 2 and Filtek). Therefore, instead of using time-consuming fracture-prone conventional incremental-fill composite, the bulk-fill composite can provide a better solution for premolar restoration in clinical practice.

## Figures and Tables

**Table 1:** Study groups with different restoration types and their descriptions.

Group name	Group description	Filling procedure
Group A	Unprepared sound teeth (positive control)	None
Group B	Prepared with an extensive Class-II cavity (MOD) but left without any restoration (negative control)	None
Group C	Restored by the universal Tetric EvoCeram (Ivoclar Vivadent and Liechtenstein)	Incremental fill (conventional)
Group D	Restored by Beautifil restorative (Shofu, Japan)	Bulk fill
Group E	Restored by Filtek posterior restorative (3M ESPE, USA)	Bulk fill
Group F	Restored by SonicFill 2 (Kerr Corp., USA)	Bulk fill

**Table 2:** Mean and standard deviation of fracture resistance of restorative premolar groups.

Groups	A	B	C	D	E	F
Mean fracture resistance (*N*)	1525.38	513.13	998.75	1172.75	817.75	921.50
SD (*N*)	161.20	69.53	138.61	171.41	85.18	64.75
*P* value	≤0.001					

**Table 3:** ANOVA of different groups of restorative premolars.

	Sum of squares	*df*	Mean square	*F*	*P* value
Between groups	4,654,866.667	5	930,973.333	61.477	≤0.001
Within groups	636,029.250	42	15,143.554	—	
Total	5,290,895.917	47	—	—	

**Table 4:** Multiple comparisons by Tukey's post hoc test.

(I) groups	(J) groups	Mean difference (I−J)	Std. error	Sig.	95% confidence interval	
					Lower bound	Upper bound
A	B	1012.250^*∗*^	61.530	0.000	828.57	1195.93
	C	526.625^*∗*^	61.530	0.000	342.94	710.31
	D	352.625^*∗*^	61.530	0.000	168.94	536.31
	E	707.625^*∗*^	61.530	0.000	523.94	891.31
	F	603.875^*∗*^	61.530	0.000	420.19	787.56

B	C	−485.625^*∗*^	61.530	0.000	−669.31	−301.94
	D	−659.625^*∗*^	61.530	0.000	−843.31	−475.94
	E	−304.625^*∗*^	61.530	0.000	−488.31	−120.94
	F	−408.375^*∗*^	61.530	0.000	−592.06	−224.69

C	D	−174.000	61.530	0.072	−357.68	9.68
	E	181.000	61.530	0.056	−2.68	364.68
	F	77.250	61.530	0.807	−106.43	260.93

D	E	355.000^*∗*^	61.530	0.000	171.32	538.68
	F	251.250^*∗*^	61.530	0.003	67.57	434.93

E	F	−103.750−	61.530	0.548	−287.43	79.93

^*∗*^Significance of mean difference is determined at 0.05 level.

## Data Availability

The test data used to support the findings of this study are included within the article.
